# Study on the propagation law of hydraulic fractures in heterogeneous permeability reservoirs

**DOI:** 10.1371/journal.pone.0328689

**Published:** 2025-07-30

**Authors:** Linhao Zou, Yinao Su, Xingsheng Xu, Wei Li, Huan Zhao, Mingxiu Zhang, Shengjie Jiao

**Affiliations:** 1 The Key Laboratory of Continental Shale Oil and Gas Accumulation and Efficient Development, Northeast Petroleum University, Daqing, Heilongjiang, China; 2 National Key Laboratory of Continental Shale Oil, Daqing, Heilongjiang, China; 3 Heilongjiang Provincial Key Laboratory of Oil and Gas Reservoir Stimulation, Daqing, Heilongjiang, China; 4 Sinopec Shengli Petroleum Administrative Bureau Co Ltd, Dongying, Shandong, China; 5 National Engineering Research Center of Oil & Gas Drilling and Completion Technology, Beijing, China; Shenyang Jianzhu UniversityCHINA

## Abstract

The development of unconventional oil and gas resources is increasingly shifting toward heterogeneous reservoirs with complex permeability distributions, making the effective control of hydraulic fracture propagation patterns critical for optimizing production. To this end, this study establishes a 3D multilayered heterogeneous reservoir model using the finite element method to analyze fracture mechanisms. The impacts of permeability heterogeneous, injection rate, and fracturing fluid viscosity on fracture morphology are systematically investigated, and the elasticity coefficient method was used to evaluate the influence weights of each parameter.The main conclusions are as follows: (1) Permeability distribution is the core factor controlling the fracture propagation direction, with HPL dominating the extension path while MPL and LPL show limited efficiency. (2) An increase in the number of permeability layers inhibits the overall expansion of cracks, and the shape of the cracks gradually changes to rectangular. (3) Higher injection rates significantly expand fracture area, whereas fracturing fluid viscosity ≥50 mPa·s stabilizes fracture morphology. (4) The elastic coefficient method identifies injection rate, permeability heterogeneous, and fracturing fluid viscosity as the key control parameters in order. This work provides theoretical guidance for optimizing hydraulic fracturing parameters in complex geological settings.

## Introduction

Under the global trend of low-carbon energy transition, the development of unconventional resources such as shale oil and tight gas has become an important direction to ensure energy security [1]. The recoverable reserves of unconventional resources are five times higher than those of conventional resources, but their development faces the dual limitations of reservoir hypotonicity and heterogeneity [2,3]. Taking the Wolfcamp Formation in the Permian Basin of North America and the Longmaxi Formation in the Sichuan Basin of China as examples, siliceous layers, clay layers, and thin carbonate interlayers are often developed within the reservoirs, and the permeability difference between the layers can be up to 10:1 or more, and a large number of fracking operations result in single-well yield loss due to insufficient fracture extension [4,5]. Therefore, revealing the fracture extension control mechanism under the multilayer permeability system is of great engineering significance for improving oilfield recovery.

Regarding studies on permeability, researchers have conducted relevant investigations [6–8]. A.N.Baykin [9] proposed a planar 3D poroelastic hydraulic fracturing model to study the effect of reservoir permeability heterogeneity on the direction of fracture extension. It was shown that the fracture extension paths were affected by permeability differences and preferentially extended in low-permeability or high-permeability layers, with stress differences playing a key role. Li [10] established a permeability-based hydraulic fracturing model to study hydraulic fracturing characteristics in heterogeneous rock materials. It was found that an increase in the fracture incidence height in layered rock decreases the fracture breakdown pressure, the tensile strength of non-fractured layers has a small effect, and large inclusions close to the injection point increase the breakdown pressure. Wang [11] developed a transient water-force coupling model to analyze the effect of seepage force on fracture initiation in hydraulic fracturing. It was found that fluid viscosity and wellbore pressurization rate significantly affected fracture initiation pressure and SF-induced circumferential stress. The effect of pressurization rate on fracture initiation pressure was significant in low-permeability reservoirs (<50 mD), whereas it was small in high-permeability reservoirs (>100 mD). In the same year Wang [12] improved the fluid-solid coupling method based on discrete elements and introduced seepage force into the model, revealing the mechanism of seepage force’s influence on hydraulic fracturing extension. It was shown that the low viscosity and low rate led to the shortening of fracture length, and the seepage force induced the fracture to favor the side with weak cementation and high permeability, and to form a complex branching network in the heterogeneous formation. Anthony Peirce [13] investigated the contraction dynamics of fractures in permeable elastic media after fluid injection cessation, and analyzed the effects of dimensionless parameters (ω and $\phi^{V}$) and on the fracture cessation time and length, and the contraction duration by building a mathematical model and introducing a multiscale tip asymptotic solution. Liu [14] established a non-uniform/asymmetric fracture condition for a multistage fractured horizontal well double porosity-double permeability model under the condition of non-uniform/asymmetric fracturing. It was found that the fracture length, height and asymmetry had a higher effect on the production capacity than the fracture spacing, which was verified by field cases.

Numerical simulation has played a critical role in exploring fracture behavior in complex formations [15–20]. Zhang [21] developed a 3D hydraulic-mechanical coupling model using the Combined Discrete Element Method (CDEM), verified its accuracy via the PKN model, and investigated the influence of bedding planes, stress differentials, and elastic modulus contrasts on fracture height growth. Tan [22] developed a numerical model of layered shale with transition zones based on the extended finite element method (XFEM) with cohesive zone model (CZM). The effects of ground stress, dip angle, tensile strength and anisotropy of the transition zone, anisotropy of the shale matrix, and injection rate on the vertical extension behavior of fractures were investigated. The results show that the presence of transition zone in layered strata can greatly hinder the expansion of the fracture height. Zhang [23] investigated the interaction between hydraulic fracture and the weak interface of shale reservoir using XFEM, and analyzed the key influencing factors of whether hydraulic fracture can realize the penetration of the layer. Mohammsdnejad and Andrade [24] combined Cohesive units with the extended finite element method to simulate hydraulic fracture extension in partially saturated and weakly porous formations, using a compensatory method to eliminate unphysical penetration of the fracture surface during fracture closure. Zhong [25] used the extended finite element method to study the interaction between hydraulic fracture (HF) and multiple weak interfaces in layered heterogeneous shale, revealing the coupling effect between neighboring weak interfaces, and the study shows that the former weak interface inhibits the fracture penetration ability due to material differences, while the latter weak interface is affected by the facilitating effect of the former interface’s precession, which significantly enhances the fracture extension across layers. Although some progress has been made in the study of fracture extension in hydraulic fracturing of heterogeneous composite formations, there is a lack of research on the mechanism of fracture extension in multilayer permeability systems.

In summary, this study investigates fracture propagation mechanisms in multilayer heterogeneous permeability reservoirs. A 3D numerical model was developed using the finite element method (FEM), specifically elucidating the influence of fracturing fluid injection rate, viscosity, and permeability distribution patterns on fracture propagation. Finally, a multi-factor analysis is carried out by the elasticity coefficient method to determine the weights of each influencing factor on fracture extension, which further improves the targeting of hydraulic fracturing parameter optimization and enhances the effect of reservoir transformation.

## Theoretical model

### Constitutive model and fluid flow model

This study aims to simulate the fracture propagation in heterogeneous reservoir hydraulic fracturing and uses cohesive units to establish a model. The specific equation is as follows [26]:


$$\sigma =\left\{\begin{array}{c} \sigma_{n}\\ \sigma_{s}\\ \sigma_{t} \end{array}\right\}=K\varepsilon =\left(\begin{array}{ccc} {}*20cK_{nn} & K_{ns} & K_{nt}\\ K_{ns} & K_{ss} & K_{st}\\ K_{nt} & K_{st} & K_{tt} \end{array}\right)\left\{\begin{array}{c} \varepsilon_{n}\\ \varepsilon_{s}\\ \varepsilon_{t} \end{array}\right\}$$ (1)

$$\varepsilon_{n}=\frac{d_{n}}{T_{0}},\varepsilon_{s}=\frac{d_{s}}{T_{0}},\varepsilon_{t}=\frac{d_{t}}{T_{0}}$$ (2)

Where, σ is the total stress borne by the fracture unit, MPa; ε is the total strain of the cohesive fracture unit; $T_{0}$ is the initial thickness of the cohesive fracture unit; $\sigma_{n}$ is the normal stress, MPa; and $\sigma_{s}$、$\sigma_{t}$ are the tangential stresses in two directions, MPa; K is the stiffness matrix of the fracture unit before damage; $\varepsilon_{n}$ is the normal strain; $\varepsilon_{s}$、$\varepsilon_{t}$ are the tangential strains in two directions; $d_{n}$ is the displacement in the normal direction, m; $d_{s}$、$d_{t}$ are the displacements in the two tangential directions, m

After injection into the formation, the fracturing fluid interacts with the fracture surface to generate driving force, which directly controls the process of fracture opening and expansion. Based on the cohesive unit model, it is assumed that the fluid flow in the fracture meets the continuous incompressible condition, which is specifically manifested in two basic modes: tangential flow along the fracture surface and normal seepage perpendicular to the fracture surface.

The Newtonian fluid injected at a constant flow rate forms a tangential flow in the middle layer of the cohesive cell, and its flow characteristics follow a specific governing equation as [27]:


$$q=-\frac{d^{3}}{12\mu }\nabla p$$ (3)

Where, q is the volume flow rate per unit area of the cohesive fracture unit, m^3^/s; d is the unit opening, m; μ is the viscosity of the fracturing fluid, Pa·s; ∇p is the tangential flow pressure gradient, Pa/m.

Fracturing fluid flows in the normal direction within cohesive units, and its filtration rate is correlated with the formation pressure variation, as expressed in [28]:


$$\begin{array}{c} q_{t}=c_{t}\left(p_{i}-p_{t}\right)\\ q_{b}=c_{b}\left(p_{i}-p_{b}\right) \end{array}$$ (4)

Where, $q_{t}$、$q_{b}$ are the flow velocities of the permeable formation, m^3^/s; $c_{t}$、$c_{b}$ are the upper and lower surface filtration coefficients, $p_{t}$、$p_{b}$、$p_{i}$ are the fluid pressures of the upper surface, lower surface and middle layer of the fluid, Pa.

### Fracture propagation criteria

In this paper, the quadratic stress criterion is used as the initial damage criterion [29]. The damage criterion can be expressed as follows: when the sum of the squares of the ratios of the stress components of the cohesive element in directions $\left\langle \sigma_{n}\right\rangle$, $\sigma_{s}$ and $\sigma_{t}$ to the stress components of directions $\sigma_{n}^{0}$、$\sigma_{s}^{0}$ and $\sigma_{t}^{0}$ the parameters in the corresponding directions is equal to 1, the element is considered to have started to be damaged and the expression is as follows:


$${\left\{\frac{\left\langle \sigma_{n}\right\rangle }{\sigma_{n}^{0}}\right\}}^{2}+{\left\{\frac{\sigma_{s}}{\sigma_{s}^{0}}\right\}}^{2}+{\left\{\frac{\sigma_{t}}{\sigma_{t}^{0}}\right\}}^{2}=1$$ (5)

Where, $\sigma_{n}^{0}$ is the critical stress in the normal direction, MPa; $\sigma_{s}^{0}$ and $\sigma_{t}^{0}$ are the critical shear stresses in the two tangential directions, MPa.

When the energy release rate of the crack tip node reaches a certain level, the connection interface of the corresponding cohesive unit will dissociate, driving the crack to extend forward [30]. The BK criterion is used to define the failure evolution of the cohesive unit, setting the fracture energy in the two tangential directions equal, and judging the complete failure of the material by the critical displacement, and the expression is as follows [31]:


$$G_{n}^{c}+\left(G_{s}^{c}-G_{n}^{c}\right){\left\{\frac{G_{s}+G_{t}}{G_{n}+G_{s}+G_{t}}\right\}}^{\eta }=G^{c}$$ (6)

Where, $G^{c}$ is the total critical energy release rate of the unit, N/mm; η is a constant related to the material, dimensionless; $G_{n}^{c}$ is the critical energy release rate of the unit normal, N/mm; $G_{s}^{c}$ is the critical energy release rate of the unit tangential, N/mm; $G_{n}$ is the energy release rate of the unit normal, N/mm; and $G_{s}$、$G_{t}$ is the energy release rate of the unit tangential, N/mm.

The cohesive unit damage evolution process adopts the stiffness degradation model, which simulates the progressive damage process of the material through linear stiffness attenuation, and the expression is as follows [32]:


$$\begin{array}{c} \sigma_{n}=\left\{ \begin{array}{c} \left(1-D\right)\overset{―}{\sigma_{n}}\sigma_{n}\geq 0\\ \overset{―}{\sigma_{n}}\sigma_{n}\leq 0 \end{array}\right.\\ \sigma_{s}=\left(1-D\right)\overset{―}{\sigma_{s}}\\ \sigma_{t}=\left(1-D\right)\overset{―}{\sigma_{t}} \end{array}$$ (7)

Where, $\overset{―}{\sigma_{n}}$ represents the normal stress of the unit when it is not damaged, MPa; $\overset{―}{\sigma_{s}}$、$\overset{―}{\sigma_{t}}$ can be the stress in the two tangential directions of the unit, MPa; D represents the dimensionless damage factor, between 0 and 1.

The damage factor is calculated using the exponential displacement criterion, and the expression is as follows [32]:


$$D=1-\left\{\frac{d_{m}^{0}}{d_{m}^{\max}}\right\}\left\{1-\frac{1-\exp\left(-\alpha \left(\frac{d_{m}^{\max}-d_{m}^{0}}{d_{m}^{f}-d_{m}^{0}}\right)\right)}{1-\exp\left(-\alpha \right)}\right\}$$ (8)

Where, α is the exponent of crack propagation rate; $d_{m}^{0}$ is the displacement when the unit just begins to be damaged; $d_{m}^{\max}$ is the maximum displacement value during the damage process of the unit; and $d_{m}^{f}$ is the displacement when the unit is completely destroyed.

### Model development and experimental validatio

#### Numerical model development.

To investigate the hydraulic fracture propagation mechanism in multi-layer heterogeneous permeability reservoirs, this study constructed a 3D model of a multi-layer heterogeneous permeability reservoir. In the model, permeability heterogeneity is achieved through a hierarchical structure with relative differences, dividing the reservoir vertically into high permeability layers (HPL), medium permeability layers (MPL), and low permeability layers (LPL). The permeability values are not set within an absolute range; instead, they are artificially calibrated based on reservoir geological data to generate permeability distribution characteristics consistent with geological statistical patterns, thereby forming a controllable heterogeneous gradient and providing clear boundary conditions for mechanism analysis.

**Fig 1:**
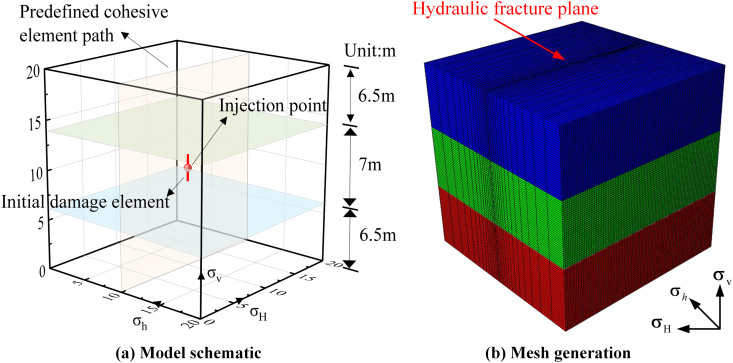
Multi-layer heterogeneous permeability 3D model. (a) Model schematic. (b) Mesh generation.

The model’s grid partitioning adopts a refined domain-based strategy, as shown in **Fig 1**. The model dimensions are 20 m × 20 m × 20 m, with an intermediate reservoir height of 7 m and overlying and underlying strata heights of 6.5 m each. Non-reservoir regions use coarser hexahedral grids (C3D8P) with a cell size of 2 m to conserve computational resources. The reservoir region employs local refinement to reduce cell dimensions to 0.5 m × 0.5 m × 0.5 m, ensuring high-precision analysis of stress and flow fields while avoiding computational redundancy from global refinement. To simulate fracture propagation, Cohesive cells (COH3D8P) are embedded along the reservoir mid-face. Permeability parameters are mapped to each grid cell based on the previously generated heterogeneous distribution. The model comprises 160,000 C3D8P continuous medium elements and 10,000 Cohesive elements. Through mesh independence verification, when progressively refined to a 0.3 m mesh size, the deviation in fracture trajectory and propagation area is less than 3%. Therefore, a 0.5 m mesh size is sufficient to balance computational accuracy and efficiency. Hydraulic fracturing simulation Injection of fracturing fluid is centered on the Cohesive layer, with a total simulation time domain of 600 s. Based on the critical stress criterion, the two cohesive elements adjacent to the injection node are activated as initial damage sources.

In this study, the rock mechanical parameters of the three layers were set to the same value, ignoring the bedding differences between the reservoir and the surrounding strata, and focusing on the expansion behavior of fractures penetrating layers with different permeabilities. The rock mechanical parameters were comprehensively referenced from the research results of He and Cong et al [33–37]. Parameters such as the geostress field, permeability, and fracturing fluid viscosity were all derived from experimental data at the oil field site. The specific reservoir and fracturing parameters are shown in Table 1.

**Table 1:** Basic parameters of multi-layer heterogeneous reservoir model.

Type	Parameters	Values
Reservoir rock	Minimum horizontal principal stress/MPa	22
	Maximum horizontal principal stress/MPa	24
	Vertical principal stress/MPa	26
	Elastic modulus/GPa	20.52
	Poisson ratio	0.22
	Porosity/%	2
	Tensile strength/MPa	6
	Tensile fracture energy/N·m^-1^	30000
	Permeability/mD	2.5-160
Fracturing fluid	Fluid specific gravity/N·m^-3^	9800
	Fluid viscosity/mPa·s	30-400
	Injection rates/m^3^·min^-1^	1-6

#### Model validation.

In order to verify the reliability of the multilayer permeability model, artificial rock samples with a size of 200 mm × 200 mm × 200 mm and three permeability layers of 5 mD:10 mD:15 mD were prepared for hydraulic fracturing experiments in this study, and the experimental results were compared with the simulation results.

**Fig 2:**
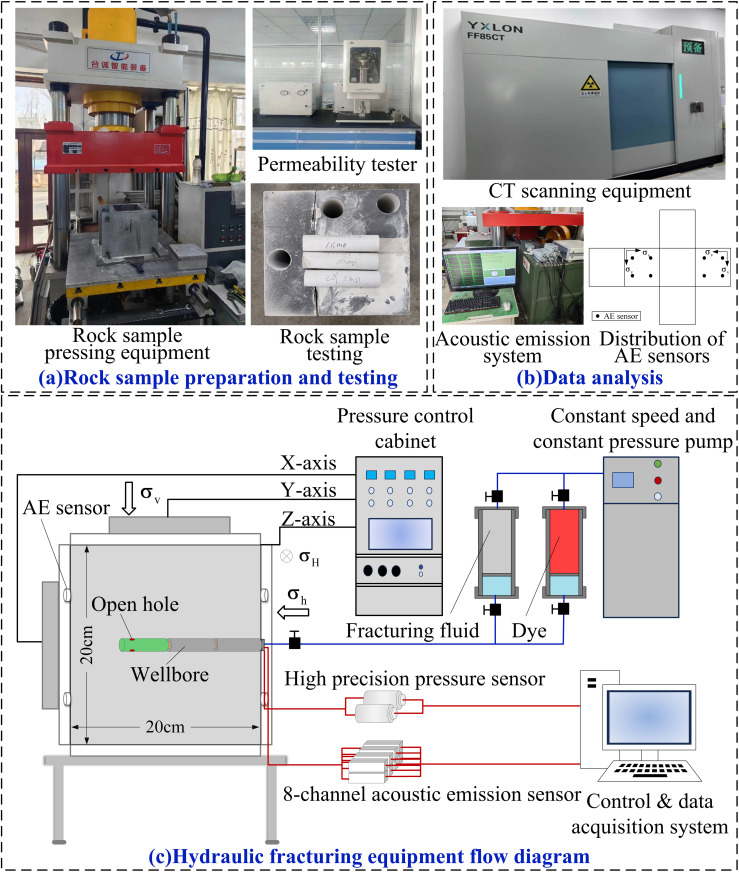
Schematic diagram of artificial rock sample preparation and hydraulic fracturing device. (a) Rock sample preparation and testing. (b) Data analysis.(c) Hydraulic fracturing equipment flow diagram.

The process of preparing multilayer heterogeneous rock samples is shown in **Fig 2(a)**. The process of artificial rock sample preparation is as follows: (1) According to the target permeability requirement, epoxy resin binder and curing agent are mixed in a preset ratio. (2) Subsequently, the mold was filled, and the mixed materials were loaded into the mold in layers, with the thickness of the three layers of rock samples being 65 mm, 70 mm, and 65 mm, respectively, and the five-clawed shovel was used to stir slightly to eliminate the internal pores after each layer was filled. (3) After filling, the rock samples were pressed and held in the hydraulic press for 30 minutes. (4) The pressed rock samples were transferred to a thermostat for 36 hours. (5) The specimens were left at room temperature for 15 days.To meet the experimental requirements, the study was based on the preparation of large-size artificial rock samples and the simultaneous stratified drilling of core columns with a diameter of Φ25 mm. The key rock mechanical parameters such as permeability, tensile strength and elastic modulus of the artificial rock samples were measured, in which the average relative error between the measured and set permeability values was ≤ 1%, which meets the requirements of API core testing specifications. All the rock mechanical parameters are in the typical sandstone mechanical characteristic interval, which can provide a reliable basis for the research. The experimental test adopts the independently constructed true triaxial hydraulic fracturing physical simulation system (**Fig 2c**).The true triaxial loading system is equipped with three independently servo-controlled hydraulic cylinders, capable of applying a maximum confining pressure of 70 MPa, and simulates the in-situ stress state of the reservoir within an integrated fluid-solid coupled chamber. The fracturing fluid injection unit utilizes a high-precision plunger pump with a flow rate range of 0.01–50 mL/min and a pressure resolution of 0.1 MPa. Combining CT scanning with synchronous acoustic emission (AE) monitoring technology, the acoustic emission monitoring system consists of an 8-channel wideband AE sensor, with an emission frequency set to 40 kHz, a sampling period set to 0.4 μs, and a P-wave wavelength of 70 mm. The acoustic emission sensors are attached to the rock sample surface, with the AE probes distributed as shown in **Fig 2(b)** “Distribution of AE sensors.” The CT scanning system employs microfocus X-ray CT technology with a resolution of 10 μm and a scanning interval of 0.1 mm. The specimen is placed at the center of the CT frame and X-ray CT scanning is performed from one side to the other. After the experiment, the rock sample is subjected to three-dimensional reconstruction, and the geometric characteristics of the cracks are quantitatively analyzed, enabling multi-dimensional characterization and comparison of crack dynamic propagation. In the hydraulic fracturing and numerical simulation process, the maximum horizontal principal stress and minimum horizontal principal stress are 22 MPa and 24 MPa, respectively, the vertical stress is 26 MPa, the viscosity of fracturing fluid is 30mPa·s, the injection rate is 20 ml/min, and the rock mechanical parameters of the numerical model are set up in accordance with the measured parameters of the man-made core.

**Fig 3:**
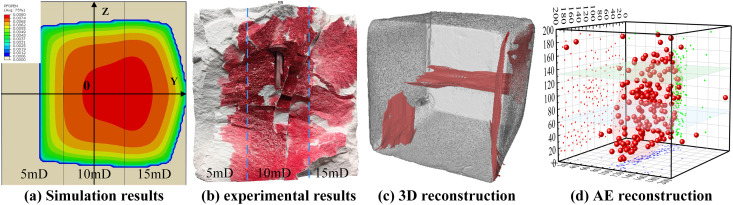
Comparison between numerical model and physical model test. (a) Simulation results. (b) experimental results. (c) 3D reconstruction. (d) AE reconstruction.

Experimental results demonstrated that fracture propagation exhibited pronounced sensitivity to permeability variation. As shown in **Fig 3(b)**, fractures extended extensively through the HPL of 10 mD and 15 mD, successfully penetrating the 15 mD layer, while propagation in the low-permeability 5 mD layer was significantly constrained. Specifically, the target reservoir section (10 mD) exhibited the largest fracture area, whereas fracture development in the 5 mD layer was substantially inhibited. In the 5 mD zone, fractures showed a nearly rectangular shape with limited extension and low complexity. In contrast, a fan-shaped propagation pattern was observed in the 15 mD HPL, with a markedly increased fracture area. In the target layer with intermediate permeability, fractures primarily exhibited a rectangular morphology, which closely matched the simulation results shown in **Fig 3(a)**. Moreover, the fracture patterns in **Fig 3(c)** and 3(d) further confirmed these trends.

**Fig 4:**
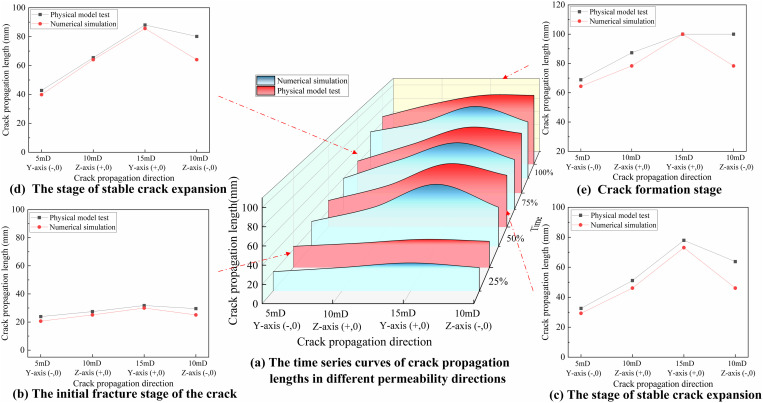
The temporal variation curves of crack propagation length in different permeability directions. (a) The time series curves of crack propagation lengths in different permeability directions. (b) The initial fracture stage of the erack. (c) The stage or stable crack expansion. (d) The stage of stable crack expansion. (e) Crack formation stage.

A comparative analysis was conducted between hydraulic fracturing physical specimens and numerical simulations using AE technology. As shown in **Fig 4**, the temporal evolution of crack propagation length varies with different permeability directions. Due to the temporal scale differences between numerical simulations and experimental studies, and the loading damage incurred during the experimental process, only a small amount of AE events is recorded before specimen fracture, and this remains largely unchanged. Therefore, the process from initial fracture to complete crack formation is divided into four equal evolutionary stages based on time: corresponding to 25% (initial fracture stage), 50% (stable crack propagation stage), 75% (accelerated crack penetration stage), and 100% (crack formation stage). The method of dividing the crack propagation direction is shown in **Fig 3(a)**. For example, the notation “10mD Z-axis (+,0)” indicates that the crack originates at the center of the specimen and propagates along the positive Z-axis in the 10mD direction. As shown in **Fig 4(b)**, during the initial fracture stage, the crack has not penetrated the 10 mD permeability layer, and AE events are primarily concentrated in the wellbore region, with crack propagation lengths being roughly consistent across all directions. As the crack progresses from the initial rupture stage to the accelerated penetration stage, the proportion of AE events increases from 26% to 73%. Additionally, the density of AE events in the 15mD permeability layer is significantly higher than in the 5mD permeability layer, indicating that energy release is more active in HPL, and cracks tend to propagate preferentially toward HPL. Experimental results show that in the physical model experiment, the crack extended along the Y-axis (+,0) direction from 35.75 mm to 88.08 mm, with a growth rate of 177%. In the numerical simulation results, the growth rate of crack propagation along the same direction was 185%, highly consistent with the experimental results. When the crack propagated to 100% (i.e., the fully developed stage), the crack propagation trends were generally consistent, with an average relative error of less than 10%, further validating the controlling role of permeability in crack propagation. Therefore, by comparing the numerical simulation results with the hydraulic fracturing experimental results, we verified the validity and accuracy of the established multilayer heterogeneous permeability reservoir model, which is able to accurately predict the extension behavior of fractures in layers with different permeabilities, and can provide a reliable theoretical basis for the actual reservoir modification.

## Numerical simulation results and analysis

### The influence of permeability

#### The influence of permeability distribution.

In order to deeply study the influence of permeability distribution on crack extension in inhomogeneous strata, this study keeps the permeability in the horizontal ground stress direction unchanged and adjusts the permeability parameters of the vertical strata. In the numerical model construction, the stratum is equally divided into layers along the vertical stress direction, and four typical working conditions are formed by setting different permeability combinations, the extreme difference of permeability of neighboring strata is denoted by $R_{i}$, and the difference of extreme difference is denoted by $\Delta \sigma_{R}=\left|R_{1}-R_{2}\right|$, as shown below:

(1) Increasing polar deviation in the case of permeability polar deviation $R_{1}=R_{2}$;
(2) If the permeability distribution is “low-middle-high” and the extreme difference is different on both sides, the difference of extreme difference is increasing;
(3) If the penetration rate distribution is “medium-low-high” and the polar deviation is different on both sides, the value of the polar deviation is increasing;
(4) In the case of a “low-high-medium” permeability distribution with different polar deviations, the value of the polar deviation is increasing.

**Fig 5:**
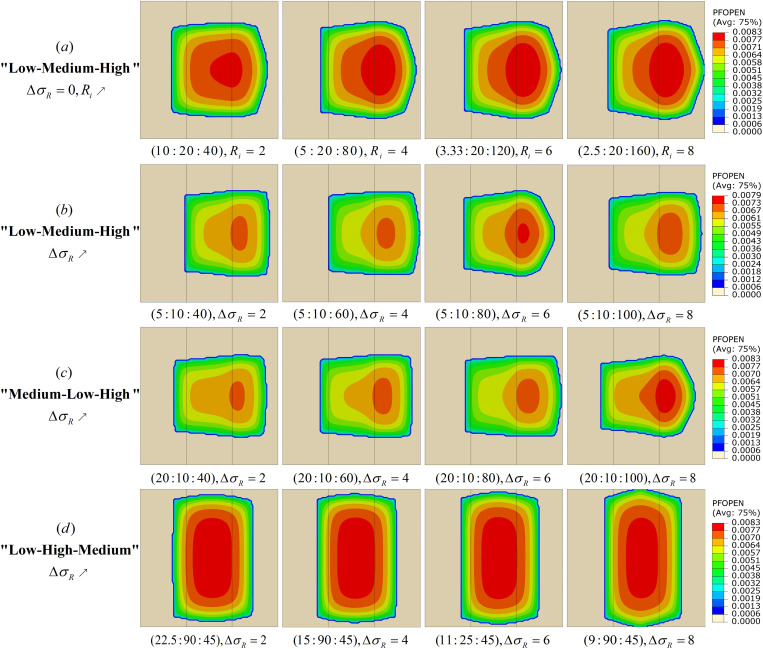
Cloud diagram of the effect of permeability distribution on fracture extension. (a) “Low-Medium-High”$\Delta \sigma_{R}=0,\;R_{i}↗$. (b) “Low-Medium-High”$\Delta \sigma_{R}↗$. (c) “Medium-Low-High”$\Delta \sigma_{R}↗$. (d) “-Low-High-Medium”$\Delta \sigma_{R}↗$.

This study systematically analyzed the crack propagation law in different permeability layers through numerical simulation methods, and revealed the influence mechanism of permeability distribution on crack geometry and propagation mode. A total of 16 groups of multi-layer heterogeneous permeability reservoir models were established, and the simulation results are shown in **Fig 5**.

As shown in **Fig 5(a)**, under a “low-medium-high” permeability distribution with symmetrically increasing contrast $R_{i}$ between adjacent layers, the fracture width exhibits a distinct stratified distribution. When $R_{i}$ increases from 2 to 8, the area where the fracture width exceeds 7.7 mm in the MPL and LPL layers gradually decreases. This phenomenon is primarily attributed to the preferential absorption of fracturing fluid by the HPL, which leads to preferential fracture propagation in the HPL while suppressing the fracture width in the MPL and LPL. As illustrated in **Fig 5(b)**, when the permeability distribution remains “low-medium-high” but $R_{i}$ on both sides are asymmetric, the overall fracture geometry becomes more rectangular. This indicates that variations in $\Delta \sigma_{R}$ affect not only the propagation direction of the fracture but also its geometric shape. As $\Delta \sigma_{R}$ increases, the fracture propagation area within the LPL expands, and the region of maximum fracture width gradually shifts toward the HPL. This shift results from changes in the fluid flow path induced by $\Delta \sigma_{R}$, due to the higher permeability in the HPL, the fracturing fluid flows more easily, concentrating fracture width within the HPL. This observation is consistent with the findings of Luo [38]. As shown in **Fig 5(c)**, under a “medium-low-high” permeability distribution, the fracture propagation length exhibits a nonlinear variation, first increasing and then decreasing as $\Delta \sigma_{R}$ increases. The maximum fracture length occurs at $\Delta \sigma_{R}$ = 6. This is caused by the dynamic stress barrier effect induced by the intermediate LPL layer, which temporarily impedes fracture growth. However, when $\Delta \sigma_{R}$ increases to 8 and the HPL permeability reaches 100 mD, enhanced fluid channeling leads to dominant horizontal fracture propagation, and the fracture width gradually increases. **Fig 5(d)** demonstrates that in a “low-high-medium” permeability distribution, $\Delta \sigma_{R}$ exerts a significant influence on fracture propagation behavior. When the permeability distribution varies from (22.5mD: 90mD: 45mD) to (9mD: 90mD: 45mD), the fracture propagation shows strong directionality, primarily extending along the HPL. This is due to the large permeability contrast between the HPL and its adjacent layers, which creates a pressure gradient that drives the fracturing fluid preferentially into the HPL, thereby enhancing fracture growth within that layer. As $\Delta \sigma_{R}$ increases, the fracture extension within the HPL becomes more pronounced, indicating that greater permeability contrast further promotes fracture propagation along the HPL.

**Fig 6:**
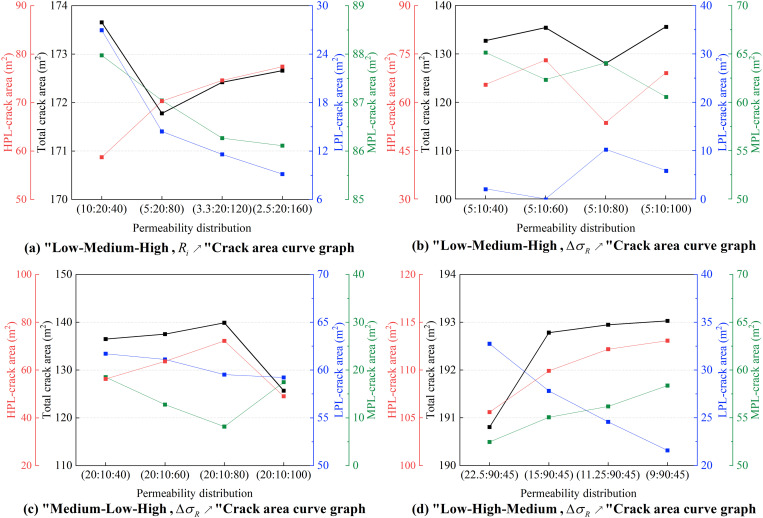
Curve of fracture propagation area under different permeability distributions. (a) “Low-Medium-High, $R_{i}↗$ ”Crack area curve graph. (b) “Low-Medium-High, $\Delta \sigma_{R}↗$ ” Crack area curve graph. (c) “Medium-Low-High, $\Delta \sigma_{R}↗$ ” Crack area curve graph. (d) “-Low-High-Medium, $\Delta \sigma_{R}↗$ ” Crack area curve graph.

As shown in **Fig 6(a)**, under the “low-medium-high” permeability distribution with increasing $R_{i}$, the fracture area curve exhibits a nonlinear trend. The area variations of the HPL and LPL display a clear symmetry, while the fracture areas in the MPL and LPL gradually decrease as $R_{i}$ increases. When $R_{i}$ increases from 2 to 4, the fracture area in HPL grows from 58.72 m^2^ to 70.31 m^2^, a 19.7% increase, while the area in LPL drops from 26.96 m^2^ to 14.42 m^2^, a 46.5% decrease. This indicates that the fluid injection effect increasingly concentrates in HPL as $R_{i}$ rises, thereby suppressing fracture growth in LPL. As $R_{i}$ continues to increase, the reduction rate of fracture area in LPL and MPL slows down, while HPL maintains a high growth rate, partially offsetting the losses in the other layers and gradually increasing the total fracture area. These results demonstrate that increasing $R_{i}$ promotes a fracture propagation pattern dominated by HPL.

As shown in **Fig 6(b)**, for a “low-medium-high” permeability distribution with increasing $\Delta \sigma_{R}$, the total fracture area reaches an abnormal low point at $\Delta \sigma_{R}$ = 6, 3.6% lower than at $\Delta \sigma_{R}$ = 2, but rebounds with a 5.9% increase at $\Delta \sigma_{R}$ = 8. The HPL fracture area peaks at 73.09 m^2^ at $\Delta \sigma_{R}$ = 2, then decreases to 53.68 m^2^, while LPL fracture area increases by 180.9%. This may be attributed to fluid channeling when HPL permeability reaches 80 mD, which increases effective stress near the fracture and reduces HPL fracture propagation efficiency. Meanwhile, the capillary force gradient at the medium-low permeability interface drives fluid migration toward LPL, but due to LPL’s limited permeability, its growth rate is only 52.9% of that in HPL.

As shown in **Fig 6(c)**, for a “medium-low-high” permeability distribution with increasing $\Delta \sigma_{R}$, the total fracture area peaks at 139.88 m^2^ at $\Delta \sigma_{R}$ = 6, a 2.5% increase compared to $\Delta \sigma_{R}$ = 2, but drops by 10.1% to 125.68 m^2^ at $\Delta \sigma_{R}$ = 8. The results indicate that at $\Delta \sigma_{R}$ = 6, the significant permeability contrast between HPL (80 mD) and MPL (20 mD) enhances vertical fluid migration, resulting in efficient fracture growth. However, when $\Delta \sigma_{R}$ increases to 8, lateral fluid diffusion dominates, forming wider but shorter fractures. The partial recovery in LPL fracture area may be related to the stress shadow effect, where rapid fluid flow in HPL generates local high-pressure zones, diverting some fluid to LPL. Due to limited permeability, however, LPL cannot compensate for the area loss in HPL, leading to an overall decrease in fracture area.

As shown in **Fig 6(d)**, for a “low-high-medium” permeability distribution with increasing $\Delta \sigma_{R}$, the total fracture area increases, but at a diminishing rate. The fracture area in MPL grows steadily from 52.47 m^2^ to 58.37 m^2^, an 11.2% increase, which is related to fluid flow path adjustments caused by the permeability contrast between HPL and MPL. In contrast, the fracture area in LPL decreases significantly from 32.74 m^2^ to 21.59 m^2^, a 34.1% reduction. This indicates that increasing $\Delta \sigma_{R}$ strongly suppresses fracture propagation in LPL. The findings show that as the permeability contrast between HPL and LPL becomes more pronounced with increasing $\Delta \sigma_{R}$, fluid preferentially flows within HPL, inhibiting fracture extension in LPL. However, excessive $\Delta \sigma_{R}$ values can restrict fluid flow in LPL, limiting further increases in total fracture area.

### The influence of the number of permeability layers

**Fig 7:**
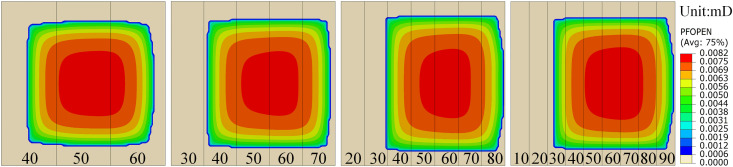
Cloud maps of the effect of multilayer permeability distribution on fracture extension.

In the previous section, we primarily investigated fracture propagation behavior in a three-layer permeability formation. However, in real geological settings, stratigraphic structures are often more complex, exhibiting multilayered permeability heterogeneity. To further elucidate the influence of permeability heterogeneity on fracture propagation, this section analyzes the fracture propagation characteristics in multilayer permeability formations. Four numerical models with varying numbers of permeability layers were constructed, featuring progressively increasing permeability values of (40: 50: 60), (30: 40: 50: 60: 70), (20: 30: 40: 50: 60: 70: 80), and (10: 20: 30: 40: 50: 60: 70: 80: 90), respectively. The model parameters were kept consistent with the three-layer model. The simulated fracture propagation results are shown in **Fig 7**.

As shown in **Fig 7**, with the increasing number of permeability layers, the fracture geometry gradually transitions from an approximately pentagonal to an approximately rectangular shape. The edges of the LPL tend to exhibit linear features, while the HPL display curved edges. Moreover, as the number of layers increases, the preferential absorption of fracturing fluid by the HPL leads to a gradual increase in fracture width and area within the high-permeability zones, while the width and area of fractures in the LPL are increasingly suppressed. The study shows that the expansion behavior of HPL is more significantly regulated with increasing complexity of the permeability distribution.

**Fig 8:**
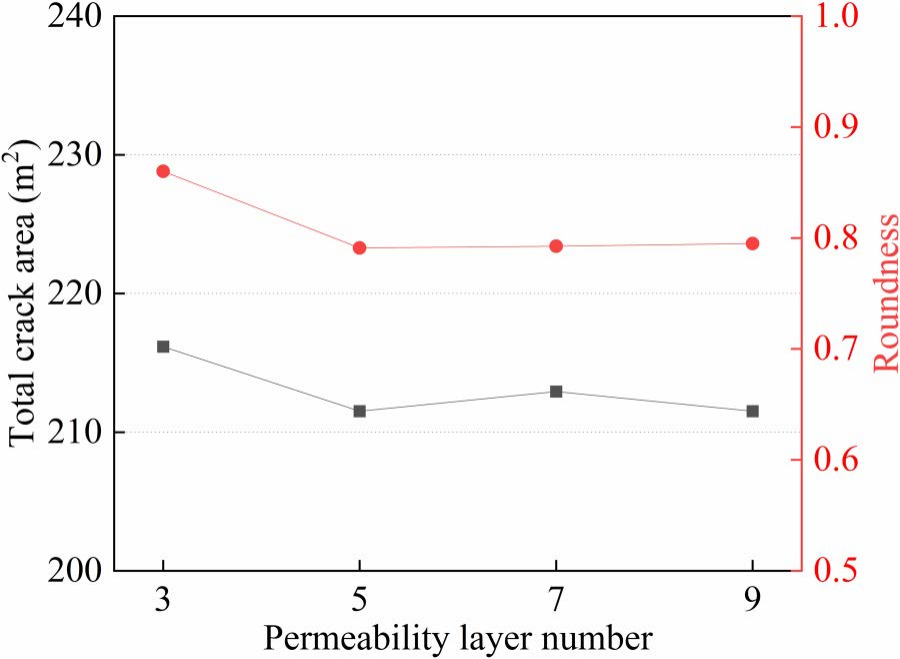
Curves of fracture propagation area and fracture circularity under multi-layer permeability models.


As shown in **Fig 8**, the curves depict the total fracture propagation area and fracture circularity for multi-layer permeability models. The study indicates that the total fracture area shows a decreasing trend and gradually stabilizes when the number of layers exceeds five. Meanwhile, the circularity of the fractures decreases from 0.86 to 0.79, with the circularity calculated using Equation (9), indicating that the fracture morphology transitions from an approximate pentagonal shape to a rectangular one. With the increase in the number of permeability layers, the complexity of formation heterogeneity is enhanced. This introduces more stress barriers in the multi-layer permeability structure, thereby restricting fracture propagation in the low-permeability layers (LPL). By comparing permeability models with different layer counts, this analysis provides a more comprehensive understanding of how formation permeability heterogeneity regulates fracture propagation, offering theoretical support for optimizing hydraulic fracturing in complex reservoirs in practical engineering applications.

$$C=\frac{4\pi A}{L^{2}}$$ (9)

Where, *A* is the area of the polygon, m^2^; *L* is the perimeter of the polygon, m.

### The influence of injection rate

**Fig 9:**
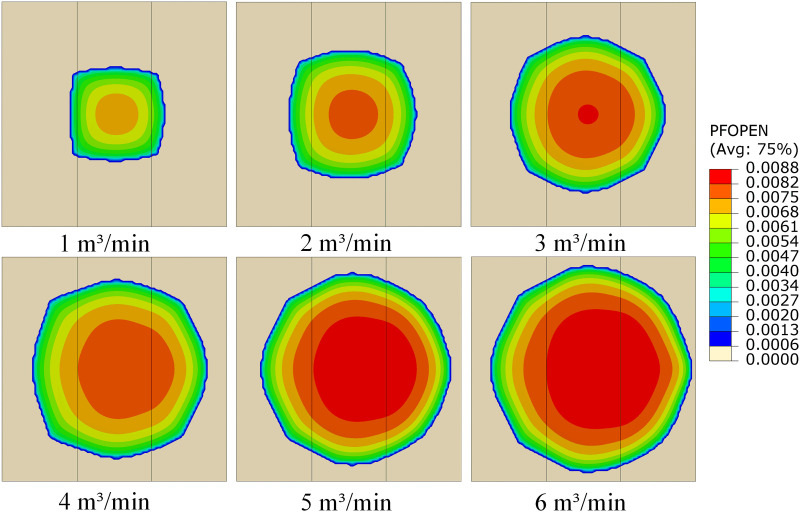
Cloud maps of fracture propagation under different injection Rates.

Fracturing fluid injection rate has a significant effect on fracture geometry, extension area, and degree of filtration loss. To investigate the impact of injection rate under heterogeneous formation conditions, a three-layer model with permeability values of 5 mD:10 mD:15 mD was established. Six injection scenarios were simulated, with injection rates set at 1 m³/min、2 m³/min、3 m³/min、4 m³/min、5 m³/min and 6 m³/min. As shown in **Fig 9** for the crack extension state, it can be found that the change of the displacement shows high sensitivity. With the increase of injection displacement, the extended area of cracks showed a significant upward trend. At the same time, the morphology of the cracks also changed significantly, and the crack morphology was close to rectangular when the injection rate ≤ 2 m^3^/min. With the increase of the injection rate, the crack morphology gradually evolved to a circle when the injection rate reached 3 m^3^/min, indicating that the influence of the heterogeneous of the formation on the crack morphology gradually weakened.

**Fig 10:**
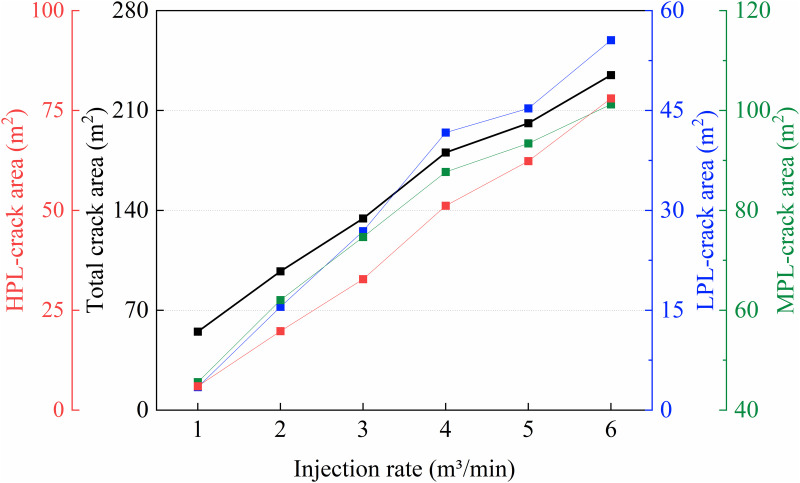
Curve of Fracture Extension Area under Different Injection Rates.

As shown in **Fig 10**, when the injection rate increases from 1 m³/min to 6 m³/min, the total fracture area increases from 55.07 m^2^ to 234.85 m^2^, a growth of 326%. Specifically, the fracture area in the HPL increases from 5.99 m^2^ to 78.04 m^2^, while the MPL increases from 45.61 m^2^ to 101.24 m^2^, a growth of 121.8%. The LPL expands from 3.47 m^2^ to 55.57 m^2^. The fracture propagation process can be divided into two stages. In the low injection rate stage (1–4 m^3^/min), MPL dominates fracture propagation, though its area proportion decreases from 82.8% to 48.6%. HPL begins to activate gradually, with its area share rising from 10.9% to 28.3%, although its growth lags behind that of MPL. LPL exhibits lower propagation efficiency in this stage, with its area proportion decreasing from 35.9% to 23.1%. This is mainly because, under low injection rates, the fluid energy is insufficient to fully activate the advantages of the HPL, and the fluid preferentially propagates through the MPL due to its moderate resistance. When the injection rate increases from 4 m^3^/min to 6 m^3^/min, the total fracture area rises from 180.55 m^2^ to 234.85 m^2^, with a slower growth rate. In this stage, HPL becomes the dominant layer, with its area share increasing from 28.3% to 33.2%. Meanwhile, the MPL growth slows, and its share decreases from 48.6% to 43.1%, with the growth slope dropping from 0.87 to 0.31. Once the injection rate reaches ≥4 m^3^/min, the fluid pressure exceeds the fracture threshold of the HPL, leading to tensile failure–dominated propagation. Fracture width increases from 6.1 mm to 8.76 mm, forming low-resistance flow channels. Therefore, to reasonably regulate the fracturing fluid injection rate in heterogeneous formations, a gradually increasing rate strategy of 3-4-5 m^3^/min is recommended. Under this approach, the fracture area growth ratio among the three layers (HPL: MPL: LPL) improves from 4.3:1.8:1 to 2.1:1.5:1, achieving a more balanced reservoir stimulation.

### The influence of fracturing fluid viscosity

**Fig 11:**
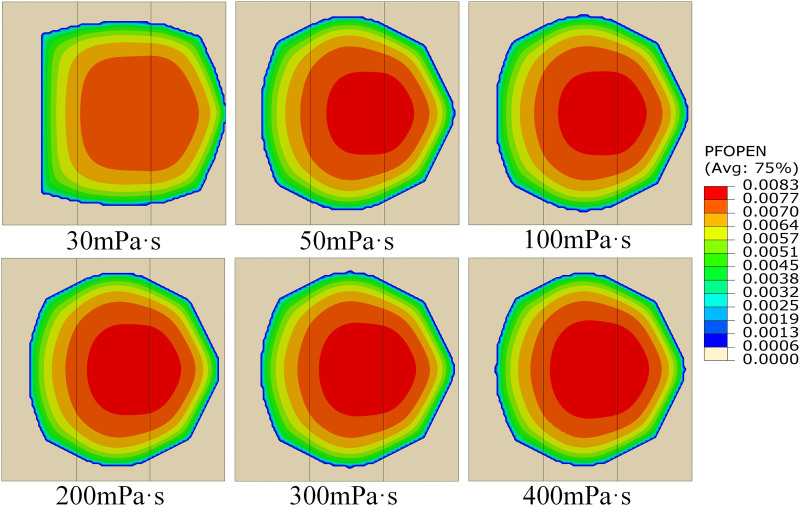
Cloud map of the influence of fracturing fluid viscosity on fracture propagation.

Fracturing fluid properties directly affect reservoir stimulation effectiveness, and precise control of viscosity parameters has a significant impact on fracture propagation, providing critical technical support for field operations. To deeply analyze the effect of fracturing fluid viscosity on fracture propagation under heterogeneous conditions, the permeability was kept at 5 mD: 10 mD: 15 mD while varying the fracturing fluid viscosity at 30 mPa·s、50 mPa·s、100 mPa·s、200 mPa·s、300 mPa·s and 400 mPa·s. **Fig 11** shows the fracture propagation status. When the fracturing fluid viscosity is 30 mPa·s, the fracture shape approximates a regular pentagon. As the viscosity increases, the fracture shape gradually transforms into an approximate regular octagon. When the fracturing fluid viscosity is ≥ 100 mPa·s, the fracture shape basically stabilizes. Additionally, observation of the fracture width distribution reveals that regions with widths greater than 7.5 mm appear only in the MPL and HPL, while none are observed in the LPL, indicating that the fracture width in the LPL is generally smaller than that in the HPL.

**Fig 12:**
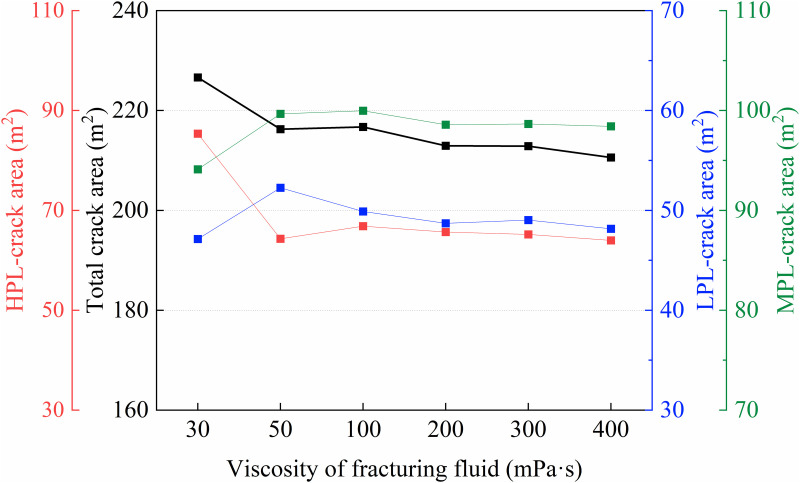
Curve of fracture extension area under different fracturing fluid viscosity.

As shown in **Fig 12**, with the increase of fracturing fluid viscosity from 30 mPa·s to 400 mPa·s, the total fracture area exhibits a gradual decreasing trend, dropping from 226.62 m^2^ to 210.58 m^2^, a reduction of approximately 7.08%. This indicates that higher viscosity fracturing fluids somewhat limit fracture propagation. When the viscosity increases from 30 mPa·s to 50 mPa·s, the fracture area in the HPL decreases significantly from 85.37 m^2^ to 64.32 m^2^, a decline of 24.65%, while the fracture areas in the LPL and MPL increase by 10.87% and 5.90%, respectively. This is mainly because the HPL, having higher permeability, allows fractures to propagate more easily at lower viscosities; however, with increased viscosity, the flow resistance of fracturing fluid in the HPL rises, and due to its high permeability, fluid loss reduction is less significant, thereby restricting fracture growth. Conversely, the moderate viscosity increase in the LPL and MPL reduces fluid loss there, enabling more energy to facilitate fracture propagation. However, when viscosity further increases from 50 to 400 mPa·s, the flow and fluid loss characteristics of the fracturing fluid stabilize under high viscosity conditions, and the influence of viscosity on fracture propagation weakens, reaching a relative equilibrium. This phenomenon is consistent with the findings of Zhao [39]. Therefore, in practical fracturing operations for multilayer heterogeneous reservoirs, it is preferable to use fracturing fluid with a viscosity of around 50 mPa·s, which allows better fracture propagation in the LPL and MPL while maintaining a certain control over the fracture area in the HPL.

### Key factor analysis

To analyze the primary controlling factors influencing fracture propagation under multilayer heterogeneous permeability conditions, this study employs the elasticity coefficient method to determine the influence weights of fracturing fluid viscosity, fracturing fluid flow rate, and permeability on the fracture propagation patterns during hydraulic fracturing. Compared to global sensitivity methods (such as the Sobol index), which require significant computational resources and large sample data sets, the elasticity coefficient method directly outputs parameter influence weights through single-variable minor perturbations at a limited computational cost, enabling precise guidance for on-site parameter optimization. Additionally, since engineering parameters often vary locally in actual fracturing operations, the local sensitivity characteristics of the elasticity coefficient method align closely with engineering operational logic. Therefore, the elasticity coefficient method was selected for sensitivity analysis. The elasticity coefficient is defined as:


$$E=\frac{\Delta Y\;/\;Y}{\Delta X\;/\;X}=\frac{\Delta Y}{\Delta X}\cdot \frac{X}{Y}$$ (10)

Where, Y is the dependent variable; X is the independent variable; ΔX and ΔY represent their respective changes. ΔX/X and ΔY/Y represent the percentage changes of variables X and Y.

After obtaining the elasticity coefficients for each factor, they are normalized to calculate the weight of each influencing factor. The weight formula is as follows:


$$W_{i}=\frac{E_{i}}{\sum_{j=1}^{n}E_{j}}$$ (11)

Where, *i* denotes the index of different influencing factors, **j* *= 1, 2,..., n, and *n* represents the total number of influencing factors.

Factors such as fracturing fluid injection rate, viscosity, and permeability play significant roles in the fracture propagation process. The calculated weights of these factors’ influence on fracture propagation are $W_{injectionrate}$ ﹥$W_{permeability}$ ﹥$W_{viscosity}$. The injection rate plays a decisive role in fracture initiation and propagation. As the injection rate increases, the fracturing fluid can more effectively create fractures in the target formation, especially in low-permeability zones, where a higher injection rate helps overcome formation resistance and forms complex fracture networks. In contrast, the influence weight of fracturing fluid viscosity is the smallest. After the viscosity reaches a certain level, the fracture propagation tends to reach a relative equilibrium state. In summary, during hydraulic fracturing of multilayer permeability reservoirs, priority should be given to ensuring sufficient injection rate to provide adequate energy for fracture initiation and propagation, while adjusting fracturing fluid viscosity to optimize fracture growth. By comprehensively considering the synergistic effects among parameters and ensuring their reasonable coordination, the optimal fracture propagation effect can be achieved.

## Conclusions

This study established a 3D finite element model of hydraulic fracturing in multilayer heterogeneous permeability reservoirs to investigate the effects of fracturing fluid injection rate, fluid viscosity, and permeability distribution on fracture propagation. Through multi-factor analysis, the weights of influencing factors on fracture extension were determined, providing targeted guidance for optimizing hydraulic fracturing parameters in multilayer permeability reservoirs. The main conclusions are as follows:

(1) In multilayer heterogeneous permeability reservoirs, hydraulic fractures preferentially propagate toward HPL. The fracture area in HPL exhibits a nonlinear increase with the enhancement of operational parameters, while LPL are significantly suppressed due to fluid competition. The extension efficiency of MPL and LPL is governed by a dynamic balance between permeability gradients and fracturing fluid leak-off, indicating that permeability differences are the key factors controlling fracture propagation direction. Therefore, in actual fracturing operations, priority should be given to improving the expansion efficiency of low LPL through engineering parameters.
(2) With increasing number of permeability layers, the total fracture extension area decreases and stabilizes beyond five layers. The fracture roundness decreases from 0.86 to 0.79, indicating a morphological transition from a near-pentagonal to a rectangular shape, Enhanced heterogeneity limits the fracture propagation in LPL. Therefore, in multilayer fracturing design, it is advisable to reasonably control the number of stratifications in the reservoir to avoid excessive layers restricting crack propagation in low-permeability strata. Additionally, it is recommended to adopt multi-stage fracturing technology to enhance overall reservoir modification efficiency.
(3) Fracturing fluid injection rate significantly affects fracture propagation. The total fracture area increases markedly with injection rate. At low injection rates, fractures primarily extend in MPL, whereas at high injection rates, HPL dominates. A stepped injection rate scheme (3-4-5 m³/min) is recommended to optimize fracture propagation across layers and achieve balanced reservoir stimulation.
(4) When fracturing fluid viscosity is ≤ 50 mPa·s, increasing viscosity leads to a decrease in total fracture area and HPL fracture area, while MPL and LPL areas increase gradually. When viscosity is ≥ 50 mPa·s, fracture morphology and extension area tend to stabilize. A viscosity of 50 mPa·s is recommended to control HPL dominance while optimizing fracture propagation in MPL and LPL.
(5) The influence weights of factors affecting fracture propagation were calculated using the elasticity coefficient method, with the results indicating that $W_{injectionrate}$ ﹥$W_{permeability}$ ﹥$W_{viscosity}$.In hydraulic fracturing operations of multilayer permeability reservoirs, priority should be given to ensuring a sufficient injection rate to provide the energy necessary for fracture initiation and propagation. Meanwhile, fracturing fluid viscosity should be adjusted to achieve balanced fracture extension across all layers.

This study provides theoretical basis and optimization recommendations for hydraulic fracturing operations in multi-layer heterogeneous permeability reservoirs, demonstrating practical engineering application value. By reasonably selecting fracturing fluid flow rate, viscosity, and optimizing reservoir modification strategies, the efficiency of fracture propagation can be significantly improved, achieving balanced reservoir modification, and providing technical support for oil and gas field development.

## Supporting information

S1 Graphs. (XLSX)
